# Narrative descriptions should replace grades and numerical ratings for clinical performance in medical education in the United States

**DOI:** 10.3389/fpsyg.2013.00668

**Published:** 2013-11-21

**Authors:** Janice L. Hanson, Adam A. Rosenberg, J. Lindsey Lane

**Affiliations:** Department of Pediatrics, University of Colorado School of MedicineAurora, CO, USA

**Keywords:** medical education, assessment, evaluation, narrative, milestones, competence, observation

## Abstract

**Background:** In medical education, evaluation of clinical performance is based almost universally on rating scales for defined aspects of performance and scores on examinations and checklists. Unfortunately, scores and grades do not capture progress and competence among learners in the complex tasks and roles required to practice medicine. While the literature suggests serious problems with the validity and reliability of ratings of clinical performance based on numerical scores, the critical issue is not that judgments about what is observed vary from rater to rater but that these judgments are lost when translated into numbers on a scale. As the Next Accreditation System of the Accreditation Council on Graduate Medical Education (ACGME) takes effect, medical educators have an opportunity to create new processes of evaluation to document and facilitate progress of medical learners in the required areas of competence.

**Proposal and initial experience**: Narrative descriptions of learner performance in the clinical environment, gathered using a framework for observation that builds a shared understanding of competence among the faculty, promise to provide meaningful qualitative data closely linked to the work of physicians. With descriptions grouped in categories and matched to milestones, core faculty can place each learner along the milestones' continua of progress. This provides the foundation for meaningful feedback to facilitate the progress of each learner as well as documentation of progress toward competence.

**Implications:** This narrative evaluation system addresses educational needs as well as the goals of the Next Accreditation System for explicitly documented progress. Educators at other levels of education and in other professions experience similar needs for authentic assessment and, with meaningful frameworks that describe roles and tasks, may also find useful a system built on descriptions of learner performance in actual work settings.

**Conclusions:** We must place medical learning and assessment in the contexts and domains in which learners do clinical work. The approach proposed here for gathering qualitative performance data in different contexts and domains is one step along the road to moving learners toward competence and mastery.

## Evaluation in medical education

Although the purposes of grading and evaluation vary across settings and teachers, we see the purposes of evaluation in medical education as gathering evidence about performance, facilitating growth of learners, inspiring excellence and making decisions about promotion within or graduation from a program. Along the continuum of pre-medical and medical education, however, grades are often used to apply ranks and they play a powerful role in determining the future of students. Grades in undergraduate college education play an important role in determining whether an applicant is accepted to medical school, and grades, board exam scores and ranks carry a great deal of weight when fourth-year medical students compete for residency placements. In fact, there is a good deal of sorting and ranking that occurs in medical schools, most of it based on examination scores and clinical grades, and there is an often-unquestioned assumption that the purpose of grading in medical schools is to sort students into groups of okay, good, and best students, and to identify the few who should not be there and dismiss them. Medical education programs that put candidates forward to compete for slots further up the professional ladder are locked into a system that requires grading, ranking and sorting as part of the application package and acceptance process; graduating medical students need grades and class ranks to get into competitive residencies. Most residency selection committees, in fact, use complicated systems based on grades and exam scores to rank graduating medical students who apply for a residency position. As a result, there is no possibility that everyone in a medical school class can excel—even if they perform excellently—because only a few can rise to the top. While there is a good deal of conversation about and effort expended toward competency-based education, underneath is the assumption that these are minimum competencies that all students or residents must meet rather than a continuum of competency levels which students or residents are expected to progress through and eventually progress beyond. Competition for grades has been described as necessary for the teaching-learning process in medical education and conventional grading systems essential for maintaining standards of teaching and learning (Lanphear, 1999). It is, therefore, hard for medical education programs to break out of this system and adopt a competency-based process that focuses on progression and final achievement rather than recording at specified intervals a summative evaluation that classifies a student based on labels. Consequently, medical education at all levels depends more upon grading schemas than on actual assessment of the knowledge, skills and attitudes that define a competent physician.

In medical education at both undergraduate (medical school) and graduate (residency and fellowship) levels, evaluation of clinical performance is based almost universally on rating scales for defined aspects of performance and scores on standardized, multiple choice examinations. While a few medical schools have adopted pass/fail grading systems, and there is evidence that the change does not affect students' future performance (White and Fantone, 2010), most use letter grades or designations of pass, high pass, honors, and fail, or even finer gradations such as high pass minus and high pass plus. Decisions about which grade to assign are usually based on points assigned for clinical evaluations, examination scores, percentile ranks and assignments (Zahn et al., 2004; Schmahmann et al., 2008). The resulting grades to a great extent determine the future opportunities of graduating medical students—whether they can compete successfully for a residency in a desired medical discipline or, indeed, any residency at all. There is, however, evidence that clerkship grades are not a reliable indicator of future performance (Takayama et al., 2006); furthermore, it is questionable whether the capabilities that are essential for good performance in medical practice are assessed with grades at all (Wimmers et al., 2008). While the hope is that the consequences of grades will motivate students to work hard and achieve excellence, grades also create a great deal of stress, anxiety and even depression for these highly-driven, success-oriented students (Rohe et al., 2006; Bloodgood et al., 2009). Most residency programs, like most medical schools, use some sort of item-based system graded on a Likert scale with descriptive anchors. The major difference from medical school is that the learner now cares less about the results of these assessments because future employment after residency is based much more on the perceived quality of the residency program nationally, letters of reference and job interviews. Even in residency, however, there is little evidence collected about actual competence in the tasks and roles that a physician must accomplish. Rather, rating scales and broad, global comments identify only a general impression of whether a resident is doing well enough to progress to independent practice.

While the evaluation landscape is slightly different in graduate medical education (GME, which includes residencies and fellowships) than in medical school in the United States, most GME program directors base their determination of success and failure primarily on a combination of Likert-scale ratings of performance in clinical settings and in-training and board examination scores. Teachers make their evaluation decisions in large part on global assessments of whether a resident or fellow is performing at, above or below the level expected for their level of training, based on their own experience in medical education and experience with residents and fellows over time. Even though agreement between medical educators who make global assessments tends to be high, whether the resident or fellow is competent is not explicitly addressed (Reznick et al., 1989; Silber et al., 2004). Furthermore, the thinking behind the rating of evaluators varies from one rater to the next and is rarely captured on evaluation forms (Govaerts et al., 2013). Similar challenges have occurred with rating scales in social work education (Regehr et al., 2007). Competence has been defined as “possessing the required abilities in all domains in a certain context at a defined stage of medical education or practice” (Frank et al., 2010) and requires specific evidence about defined capabilities. However, in our current system if a resident or fellow does not come to the attention of the program director as having difficulty it is usually assumed that they are competent in a broad range of specific skills even in the absence of explicit evidence to support that conclusion. Likewise teachers often assume that the Likert scale ratings they assign to learners have meaning beyond a global assessment. Familiarity with this approach tends to make it feel comfortable. Comments about a learner's performance are often appended to the rating scales and global assessments, and program directors or clerkship directors at the medical school level often find the comments more informative than ratings and rankings (Guerrasio et al., 2012). Verbal descriptions that teachers convey to program directors or clerkship directors but are unwilling to write down may provide even more insightful information, especially if a teacher has concerns about a learner but does not want to put something in writing that may hurt a learner's future career (Canavan et al., 2010).

In the big picture, medical educators take their jobs very seriously when it comes to grading and making decisions about whether medical students, residents and fellows are ready to graduate. They have a responsibility to society to ensure that graduating medical students are ready to care for patients in the supervised settings of residencies, and that graduating residents and fellows are ready to care for patients independently. The trust of patients, families and society as a whole depends on their taking this responsibility seriously. Accrediting organizations oversee the educational programs to add further assurance that this trust is well-founded.

In part because of this responsibility, there has been a great deal of discussion for many years in medical education about “competency-based education.” Accrediting organizations are placing increasing emphasis on defining and describing the competencies that physicians must acquire and on requiring educational programs to provide explicit evidence that medical students, residents and fellows have acquired adequate competence to practice medicine and warrant the trust of patients. In 2012 the Accreditation Council on Graduate Medical Education (ACGME) introduced the Next Accreditation System; at the beginning of the 2013 academic year the ACGME will require many programs to provide explicit evidence of competence in six broad areas (patient care, medical knowledge, communication and interpersonal skills, professionalism, practice-based learning and improvement and systems based practice) (Nasca et al., 2012; ACGME, 2013). Rating scales, points, examination scores and grades are unlikely to provide the data needed to meet this need (Squires, 1999). Our current evaluation systems do not promote, facilitate or ensure that information to support an accurate assessment of a learner's capability is collected, synthesized and applied within the educational process. Furthermore, descriptions in the literature of workplace-based assessment programs that are competency-based describe the difficulty of implementing this approach in medical education programs (Ross et al., 2011).

## Challenges of numbers and scales

In 1999 the ACGME introduced the six broad areas of competence that now guide teaching and assessment in most residencies and medical schools (Swing, 2007). These competencies, however, describe abstract skills that are challenging to identify in the context of clinical work (Balmer et al., 2009; Lurie et al., 2011) and are inadequately assessed using global rating forms (Silber et al., 2004). Most rating forms identify sub-competencies to grade within each of the six competencies and use behavioral anchors to guide assignment of a number for performance. Faculty members struggle with the process required to make the judgments necessary to assign numbers in each competency and subcompetency because they have to repeatedly navigate a complex series of steps: observation, recollection, mapping, synthesis, translation, and number assignation (See Table 1). (This description of the steps required to assign a number to a learner is derived from the authors' experiences with grading students and residents using standard evaluation forms based on the ACGME competencies over more than a decade). This process is practical and possible only if faculty members can focus and make specific observations, record observations for future synthesis and become sufficiently familiar with the competencies and sub-competencies to synthesize, translate and assign numbers accurately. There are also indications that the framework of the six ACGME competencies is counter to that which experienced clinicians use when assessing learners. Ginsberg identified eight major areas–knowledge, professionalism, patient interactions, team interactions, systems, disposition, trust, and impact on staff–that faculty use to conceptualize performance; Kennedy found that faculty use three concepts (discernment, conscientiousness, and truthfulness) and clinical skill to make judgments; Hamburger found that faculty who watch a patient encounter focus on the content and process of the encounter, patient-centered attitudes and behaviors, and interpersonal skills; Pangaro has developed and studied a four-part framework (reporting patients' data, interpreting data, managing care and educating self, patients and colleagues) for describing and evaluating the work of physicians (Pangaro, 1999; Kennedy et al., 2008; Ginsburg et al., 2010; Hamburger et al., 2011). None of these frameworks meld easily with the ACGME competency framework used on standard evaluation forms. This means that faculty members have to try to fit the round peg of their observations and judgments into the square holes of an evaluation form, often resulting in meaningless ratings and comments entered only for the purpose of getting the evaluation task done. In many cases, faculty rely more on their overall feeling about a learner, thinking, “I know a good one when I see one.” When questioned, however, few faculty members can articulate the behaviors that describe a “good learner.”

**Table 1:** **The evaluation process using numerical rating scales**.

**Evaluator works with learner in clinical setting**	**Observation**	**Recollection**	**Matching**	**Synthesis**	**Translation**	**Number assignation**
	Evaluator observes learner behaviors	When evaluation form arrives evaluator recollects instances of learner's clinical behaviors	Evaluator matches behaviors to competencies and sub-competencies	Evaluator synthesizes the recollected instances of behavior to mentally represent the learner	Evaluator translates the mental representation to the anchors and numbers	Evaluator assigns a number

While medical educators tend to agree on broad, global ratings of whether a learner is at, above or below the “expected level of performance,” agreement on scale scores for more defined areas of performance tends to be much lower. Differences in interpreting observed data and assigning numbers on a rating scale lead to lack of correlation between the numbers assigned by different raters and makes it impossible to produce reliable summative evaluations of learners that represent specific capabilities and weaknesses (Gingerich et al., 2011). Even linking the numbers on the scale with specific descriptors does not seem to improve the accuracy of raters (Regehr et al., 2007). In medical school the result is significant grade inflation, a large (almost 50%) number of faculty who believe that incompetent students are not identified and course directors who believe that students who should have failed are given a passing grade (Guerrasio et al., 2012). In residency, there is a significant halo effect with ratings correlating more with level of training than actual skills observed.

Observations and judgments are essential for the evaluation process, but in the current system we believe that they are not being used in the right way. The critical issue is not that judgments about what is observed vary from rater to rater but that these judgments are lost when translated into numbers on a scale. Methods to capture the contextual judgments of learners by their teachers must be developed (Regehr et al., 2012). Indeed, the concept that many of the capacities required for the work of a physician, such as professionalism and empathy, are social constructs that do not solely depend on the skills of a single individual but instead on interactions between individuals during patient care, has been overlooked in the drive to use psychometric tools for individual assessment (Govaerts et al., 2007; Kuper et al., 2007). If assessment in medical education is to capture these socially constructed skills we must adopt a different approach. We need to decrease or eliminate the use of numeric scales, while using methods more akin to the ethnographic approach of qualitative research to capture meaningful data in the context of clinical care, where the work of a physician occurs. Developing a shared understanding across teachers of what is expected and what is observed seems to be critical to using subjective judgments, (Gaglione et al., 2005) as is using a framework that is mentally carried into the clinical environment and reflects the work of a physician (Espey et al., 2007; Dewitt et al., 2008). Use of such conceptual frameworks in medical education is increasing, (Hemmer et al., 2008; Pangaro and ten Cate, 2013) and there is some indication that using a shared conceptual framework may even improve faculty agreement on the number assigned to a specific learner's performance (Ander et al., 2012). Frameworks that consist of rich, narrative descriptions of levels of learner performance that faculty use to match to real learners they work with also seem to help with evaluation (Regehr et al., 2012).

## The challenge of fragmented time

In times past, medical students, residents and fellows spent long periods of time working under the guidance of a consistent group of mentoring senior physicians. In the health care system of the twenty-first century, however, this occurs much less often. Medical students and residents, in particular, spend a few weeks or a month in most rotations; oftentimes the attending physicians in those settings vary on a daily or weekly basis. The apprenticeship model has broken down as the time needed to observe, assess, guide and evaluate the progress of learners has become progressively limited (Albanese et al., 2008). Many clinical faculty members are reluctant to participate in the process of assigning a number rating unless they have spent a significant amount of time, usually at least a week, with a learner. In most training environments in the United States today it is the exception rather than the rule for faculty and learners to spend extended and contiguous amounts of time with each other. Instead faculty experience with learners is fragmented and interrupted and faculty members often relate to multiple learners of different levels during their clinical work. Even though the clinical environment must be the source of all meaningful performance data because this is where the work of a physician is done, and both learners and faculty are immersed in the work milieu where the competencies and real life examples that illustrate capability of individuals are continuously present, most of these data are ignored, never discussed and rarely captured (Balmer et al., 2009). Although many tools are available to facilitate direct observation and feedback in the clinical setting, (Kogan et al., 2009) the challenge is to develop a practical process that allows faculty evaluators to sample and make sense of performance data in a complex clinical care environment and to transmit that information to program directors (Govaerts et al., 2007).

## Where is the data for meaningful feedback for learners on the path to competence?

Even with perfect inter-rater reliability, numbers and grades capture nothing specific about the performance of the particular learner and little feedback is offered to guide progress. Most rating forms include sections for written comments, but they are often not used at all or are populated with broad statements such as “good job” or “average performance” (Lye et al., 2001; Canavan et al., 2010). These comments are an indication that most evaluators use an overall impression or gestalt when completing evaluation forms rather than the stepwise analytical process that is necessary for reliability and validity. Furthermore, evaluators in medical education are reluctant to use the lower end of the rating scale or to write down negative comments, contributing to grade inflation (Speer et al., 2000; Pangaro et al., 2005). Program and clerkship directors therefore find it difficult to identify areas of performance that need support or learners who need remediation, and learners do not know which performance areas they should work on to improve.

Final grades in medical school are usually assigned by the program and clerkship directors from multiple graded components (Metheny et al., 2005) such as examination scores, Objective Structured Clinical Examinations (OSCE) (where learners rotate through a series of clinical cases with trained actors called standardized patients portraying real patients and are rated on how well they meet checklist items of various aspects of performance), and clinical ratings. This amalgamated scoring system introduces further difficulty in interpreting an individual student's strengths and weaknesses based on their grade. Furthermore, even though there is evidence that performance on a standardized medical examination with multiple choice questions early in training is a predictor of performance on similar examinations later in training (Gonnella et al., 2004) these measures do not match the end product of what a physician needs to know and do to successfully take care of patients (Harris et al., 2010).

## What can we do?

Innovators in medical education have begun to call for a radical shift from a focus on numbers and grades to a focus on narrative description (Pangaro, 1999; Govaerts et al., 2007), with a few suggesting that we do away with grades altogether and base evaluation solely on description (Hodges, 2012). Over time the descriptions of a learner begin to paint a complete picture and individual faculty who make many observations and provide many descriptions are in a position to make judgments about a learner based on thoughtful compilation of multiple data points. This requires a paradigm shift in the way we think about evaluation. We currently approach evaluation within units of clinical attachment—the block rotation—and often equate time spent on a rotation in a specific clinical discipline area with acquisition of competence in the management of medical problems in this discipline (Hodges, 2010). This “tea-steeping” approach to competence based on time spent is clearly fallacious. We must shift to thinking of evaluation and the certification of competence as based on performance and accruement of data over time, using multiple sources (Govaerts et al., 2005). We need to evaluate samples of performance in clinical work situations in such a way that we have the evidence necessary to validly state with assurance that the learners will perform similarly in future clinical work settings (Iobst et al., 2010).

One framework that has been proposed and may help medical educators achieve this evaluation goal is that of Entrustable Professional Activities (EPAs). EPAs are authentic, broad clinical tasks that residents routinely perform and that collectively describe what a resident in a specific discipline must be able to do in order to practice independently; an example relevant to pediatric and family medicine residency education would be care of the normal newborn infant (ten Cate et al., 2010). Entrustment requires observation of concrete clinical activities related to the EPA and leads to statements of awarded responsibility or, put more simply, “You are now allowed to do X without supervision.” Mulder describes using EPAs in a competency-based evaluation project in a neurology physician assistant program where developmental and attainments portfolios, progress interviews and observation data inform supervisors who make decisions about whether the learners can be entrusted with the clinical activities performed by physician assistants in neurology (Mulder et al., 2010). The idea is that supervising physicians who have worked alongside a learner use their own data, data collected by others and group consensus to make a decision about whether the learner can be trusted to perform a particular clinical activity independently. EPAs for pediatrics have been written and nested into the ACGME competencies but not comprehensively developed nor fully related to other assessment frameworks (Jones et al., 2011).

The ACGME along with the various specialty boards in the United States has developed a set of milestones frameworks for competency-based evaluation. Milestones describe five positions along a developmental progression from novice to mastery for the various sub-competencies within the six broad ACGME competencies. As part of the Next Accreditation System, beginning in July 2013, the seven disciplines that first developed milestones for residencies (diagnostic radiology, emergency medicine, internal medicine, neurological surgery, orthopedic surgery, pediatrics and urology) will be required to report on selected milestones for learners in their residency programs and to provide data to support each resident's progress toward mastery (Nasca et al., 2012). All other residency disciplines will be included in this process beginning in July 2014, and fellowships will participate over time as milestones are developed for the various subspecialties or the subspecialties develop ways to supplement the milestones of the core disciplines with relevant EPAs (ACGME, 2012). Although milestones have been described in eight disciplines (the seven noted above plus family medicine), research is still needed to determine which positions on the progression are acceptable for transition from medical school to residency to fellowship to specialty practice and from residency to general practice in a discipline for those who do not do a specialty fellowship (Hicks et al., 2010; ACGME, 2013).

## Narrative evaluation as an alternative to grades and numerical rating scales

The literature suggests that narrative evaluations may provide a useful approach to evaluation, particularly with faculty development and a shared framework for writing comments (Pangaro et al., 2005). As we seek ways to document progress toward competence using clinical performance as the basis for documentation, narrative descriptions written by faculty members who work with medical students or residents in actual clinical work settings will provide the meaningful qualitative data needed for documentation. Previous research demonstrates that clinical faculty agree on ranking of standardized narratives (Regehr et al., 2012), which suggests that faculty members share an understanding of the meaning of the narratives. Narrative comments about learners have low correlations with traditional measures of academic success, such as exam scores (Hoffman et al., 2009), suggesting they capture something the exams do not. As already mentioned, research also shows that a framework improves descriptions of learners and improves the usefulness of feedback and that descriptive methods can lead to reliable and valid evaluations (Hemmer and Pangaro, 1997; Battistone et al., 2002; Dewitt et al., 2008; Driessen et al., 2012). Descriptive comments about learner performance in clinical settings reveal both strengths and weaknesses of each learner, providing the information needed for remediation as well as facilitation of progress. These points taken together suggest that, rather than more detailed numerical scales or combinations of scores, narrative descriptions of performance in actual work settings will best help us make decisions about the competence of medical learners in the area of practice described by the milestones and the EPAs.

When deciding on a program in which to implement and study a narrative approach to competency-based assessment in medical education, the residency program in pediatrics seems a good place to begin. Although the same competencies and sub-competencies are generally used for medical school (undergraduate medical education or UME) and for residency education (graduate medical education or GME) there are significant differences between the structure and function of the educational process. Learners at the GME level are expected to take on patient care responsibilities, and are embedded in the same institution for several years, and therefore represent a population with which it is possible to introduce and study a new method of evaluation. Furthermore, as noted earlier, residents care less than medical students about the results of assessments that use rating scales and scores, because employment after residency depends more on the perceived quality of the residency program nationally, letters of reference and job interviews. They are likely to care a great deal, however, about descriptive comments that lead to feedback that will help them progress toward competence, independence and excellence. Finally, the next accreditation system places requirements on residency programs to document progress more explicitly than in the past, creating a need for new approaches to evaluation. We think, therefore, that residency education programs provide a setting in which innovation in evaluation can lead the way to meaningful change in medical education.

## A new model for evaluation

**Figure 1:**
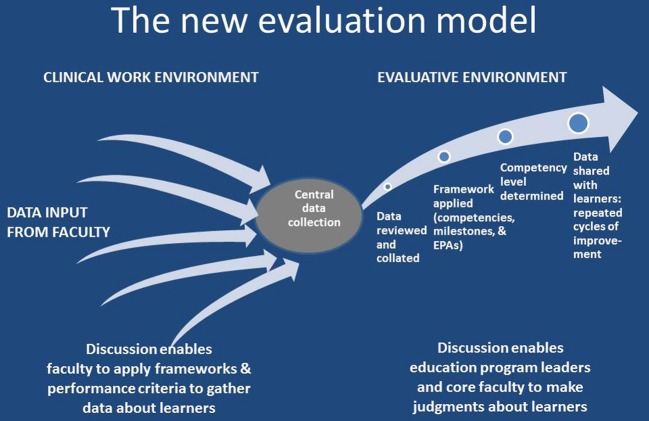
**Sample evaluation model using narrative data**.

In the Department of Pediatrics, University of Colorado School of Medicine we are developing a new model for evaluation in medical education that relies primarily on descriptive data about learner performance. We are supplementing, and hope one day to replace, the quantitative data of rating scales with the qualitative data of narrative description about learners, gathering qualitative field notes in clinical settings, much like qualitative researchers gather observation field notes. Physician faculty members write descriptions of learners' clinical performance based on direct observation of the learners in the course of physicians' daily clinical work. We have adapted the reporter/interpreter/manager/educator (RIME) framework (Pangaro, 1999) for observation and description based on the roles of physicians, the medical context and the task, and how faculty are known to conceptualize performance of the learners they supervise in the clinical environment (Kennedy et al., 2008; Ginsburg et al., 2010). (See the Supplementary Material for a copy of our descriptive comments form). This provides a meaningful, integrated framework for evaluation closely related to clinical practice. In addition, the sub-points under the major roles encompass all of the described pediatric milestones (ACGME and ABP, 2012). Faculty are asked by the learner's program to complete descriptive comments forms and record short, specific descriptions of what they saw the learner do. In contrast to what faculty are typically asked to do when they complete standard evaluation forms, the descriptive comments approach does not require faculty to place learners in a certain position in the milestones or assign a number on a rating scale with descriptive anchors but only to supply the raw data so that the program personnel can synthesize the data points and match them to the milestones. Once all available comments about a particular learner have been matched to the Pediatric Milestones, reading the set of comments matched to a particular milestone enables a faculty member who has become familiar with the developmental positions for that milestone to place the learner along the continuum of progress. The matched comments serve as qualitative data that describe the progress of a particular medical learner in relation to the expected progression, enabling the identification of learners who are not progressing adequately as well as those who are accomplishing the necessary levels of competence. This builds a picture of each learner's performance along each milestone over time and enables us to provide detailed feedback to each learner about their progress on a continual basis (See Figure 1). Knowledge-based examinations are still necessary to meet the requirements for licensing and certification, but they address only acquisition of knowledge, not whether the learner can apply knowledge to the work of a physician. Descriptive comments gathered in the context of clinical work allow program directors, who must certify that learners perform capably in all six areas of competence, to do so with more confidence.

## Challenges, concerns and resistance

Challenges with this new approach to evaluation include overcoming resistance to change, building a culture of feedback, building a shared understanding among faculty about the framework for observation and devising an efficient system to manage the qualitative data. We are using a systems approach (Littlefield et al., 2005) and undertaking considerable faculty development to move to a system of assessment based primarily on qualitative comments and feedback and away from one based on an arbitrary grading scale. Although most faculty members are familiar with the six ACGME competencies, there is much work to be done to bring them up to speed on the RIME framework and the pediatric milestones as well. Unfortunately, some faculty members think it is easier to assess a learner in the six ACGME competencies on a “grading scale,” as it does not take much time to quickly check six boxes whereas providing thoughtful comments and feedback to a resident takes more effort and may be hard to fit into a busy clinical schedule. In addition, faculty members are often uncomfortable addressing areas in need of improvement and providing strategies to make the improvement possible. When faculty members do write comments, they do not always have a shared understanding of the vocabulary used for descriptions, and they do not always provide actual descriptions of work they observed in a clinical setting. We need to create additional forums for conversation about the usefulness of descriptive comments and the clinical activities that “fit” in the role designations of the RIME framework and the pediatric milestones. We also need to determine whether a qualitative method based on descriptive comments will generate enough data to document learners' progress in all of the milestones.

The other half of the equation is the learner's interest in being assessed and in seeking meaningful feedback. Although most residents verbally express a desire for such feedback, they do not always respond with appreciation to feedback that is not all positive (Boehler et al., 2006). In addition, once they enter a residency program where there are no longer “grades,” the motivation to be assessed diminishes. Thus, a culture change involving both the evaluators and the learners needs to take place.

The management of the qualitative data needs attention but is less of a concern. It will be a fairly straightforward process to take rich qualitative feedback and match them to the milestones. The methods used for qualitative data analysis in research and evaluation apply quite well here. Core faculty members who become familiar with the pediatric milestones can quite easily read a comment and match it thematically to one or more milestones. Once the matching is accomplished, a focused picture of the resident in relation to a particular milestone emerges from a group of comments that addresses a particular aspect of clinical work. The challenge lies only in developing the logistics of a system in a way that makes the tasks of matching and selecting a developmental position for the milestone as quick and efficient as possible.

## Implications for education in other professional disciplines:

Educators in many disciplines and across the continuum of education from kindergarten through high school and on through college and graduate school struggle with issues of meaningful assessment and its relationships to learning and performance. Public school educators began working toward “outcomes based education” in the 1980s. Now that this movement has progressed to frequent standardized testing and accountability measured by standardized test scores and norm references, concerns are being raised about whether we have lost meaningful assessment and learning in the midst of test preparation, test taking and the emphasis on scores. A prominent principal in the New York City Schools recently expressed dismay about the regression of teaching about the Common Core due to standardized assessment driving teaching of splinter skills and bits of knowledge that have been divorced from their meaningful context (Burris, 2013). At the undergraduate level, one area of current controversy is whether standardized test scores and grade point averages provide the best criteria for undergraduate admissions decisions, particularly when considering students from diverse backgrounds (Jaschik, 2013). Educators share concerns about the limitations of test scores and grades but have struggled to find criteria based on narrative descriptions that predict success as well as grades do. There is trouble with grades but also with the alternatives, as we find that grades predict grades but we do not know what predicts competence.

At the graduate school level, many professions face a quandary similar to that of medicine when seeking meaningful assessment of competence. Counselors, teachers, therapists, social workers, lawyers and clergy all perform complex tasks in their work situations. Much like physicians, professionals in these areas gather information about the people they serve or care for, some information fitting into patterns and some conflicting, and they synthesize the information to make decisions about the needs of their clients or patients or students, then use the information to plan interventions or approaches to care or education or service. Educators who work with learners in these professions share the challenge of finding a framework for observation and assessment and making sense of disparate assessment data from work settings. Each profession needs meaningful frameworks that describe roles and tasks and a source of meaningful data to demonstrate progression toward competence in the work of that profession.

All education would benefit from conversations about these frameworks and a collaborative model that enables us to work together for the good of our learners, a model that helps teachers identify individual learners' strengths and weaknesses and ways to move them toward competence. Together, as a broad community of educators, we are seeking authentic assessment that is based on trusting teachers to make observations in real-life settings of learning and working, to gather data that makes sense in the context of those settings and to make judgments about the educational meaning of the observations on the path of each learner's progress. If professional education is to avoid following the path from outcomes to isolated skills and bits of knowledge that has plagued public school education, we must find our way to this authentic, contextual, meaningful assessment based in the setting where our work occurs.

A model that is the antithesis of that adopted in the United States public education system has succeeded in producing students who graduate from secondary school not only scoring highly on a final standardized test but also well prepared for entry into the workforce or higher education. This model, which forms the basis for Finland's education system, trusts the judgments of the teachers and supports them in creating curricula and lesson plans that match their students' needs. Frequent testing to gauge and analyze students' ability to respond to questions related to a common core of topics has been replaced by a long-term approach to the development of each student's capability in broad areas. Finnish teachers and schools, unlike those in the United States, are not judged annually based on the standardized test scores of their students, which allows them to practice their profession and focus their teaching to maximize the intellectual growth and development of their students. Teachers create their own tests to assess learners and use assessment results to build teaching activities that meet the needs of their learners, with just one summative, standardized examination at the end of secondary school—much as we propose here for medical education (Heilig, 2013).

## A way forward to the future: needed research and program development

There is, of course, much work to do to develop the practical approaches needed to fully implement narrative-based evaluation programs in the many contexts of medical education. Once practical approaches have been developed, medical education researchers need to study the process of sampling performance data to determine what to sample, when to sample, how much to sample, when to repeat sampling, and what observations generalize or transfer to other clinical settings. Future researchers must gather evidence for the validity of the milestones, which are constructions of progression through the work environment that have not yet been tested for assessing progression through the work environment. Although the milestones were created from the experience of experts, we need evidence about how well they meet educational needs in various medical education settings. Finally, researchers must also gather evidence for the validity of the process of grouping narrative comments according to milestones and then making judgments about which of five described positions on a continuum best matches a set of comments. Intuitively, this process of grouping and matching makes sense because the comments that compose the data come from the actual work environment and the continua of the milestones have been written to reflect the work environment, but this does need to be studied.

## Concluding thoughts

As is true in many fields of education, the focus on grades and scores on frequent standardized tests is alive and well in medical education in the United States (Rosemartin, 2013). Unfortunately, this distracts learners from focusing on what is important to become a good physician. Like many other fields, medical knowledge has grown exponentially and it is now impossible to know or remember everything one needs to practice. Medical schools have introduced courses with early exposure to clinical work environments, interdisciplinary learning and other curricular components to try to provide scaffolding on which medical students can hang the information they must learn. Full integration of a student's learning is hampered, however, when they are driven to view these courses as irrelevant to their need to excel on examinations that test primarily knowledge. Medical students and residents need a foundation for a lifetime of clinical work: excellent basic clinical skills in gathering all necessary data, discernment of their own limitations, and a conscientious approach that drives them to figure out each patient's problem and seek out those who can help (Kennedy et al., 2008). We must place medical learning and assessment in the contexts and domains in which our learners to their clinical work. The approach proposed here for gathering qualitative performance data in different contexts and domains in residency is one step along the road to moving our learners toward competence and eventual mastery.

### Conflict of interest statement

The authors declare that the research was conducted in the absence of any commercial or financial relationships that could be construed as a potential conflict of interest.
