# Supplementary material for: Narrative descriptions should replace grades and numerical ratings for clinical performance in medical education in the United States
Source: Front Psychol. 2013 Nov 21;4:668. doi: 10.3389/fpsyg.2013.00668 (PMC3836691; doi:10.3389/fpsyg.2013.00668)
Supplement: Supplementary file 1 [file DataSheet1.DOCX]

| **Professional roles for comment:** | **Please write your comments here:** |
| --- | --- |
| **Roles: Reporter/Interpreter:**   - Listens carefully - Gathers information (interview, physical exam, labs, records, radiographic studies) - Synthesizes data - Asks questions - Applies knowledge - Develops and prioritizes diagnoses - Presents data and interpretations   **Role: Manager of care:**   - Builds trust - Negotiates - Plans treatment - Writes notes - Communicates with patients and families - Works with team   **Role: Manager of systems:**   - Refers/consults - Coordinates care - Transfers care - Identifies system problems & solutions   **Role: Educator:**   - Shares information with patient/family - Teaches colleagues   **Role: Leader:**   - Advocates for patients - Advocates for populations - Provides role model - Leads team - Supervises | 1. ***Note the context* (setting, clinical situation, special circumstances, learning goal):** 2. ***Write what you saw* the student, resident or fellow do:** 3. **Record *key feedback points* (areas for learning and improvement and areas of strength):**   **1.**  **2.**  **3.** |
| **Role: Professional (foundations of practice):**   - Organization, time management - Discerns limits and appropriate roles - Conscientious, positive attitude - Truthful, trustworthy - Acquires and applies knowledge - Takes responsibility for education - Develops learning goals - Humanism, compassion, empathy - Integrity, respect - Duty, accountability; responsiveness to patients supersedes self-interest - Manages stress, seeks help appropriately, balances work and life, adjusts to change - Handles ambiguity/uncertainty well |  |
